# Enhanced Lateral Resolution in Acoustic Imaging: From High- to Super-Resolution

**DOI:** 10.3390/s26061992

**Published:** 2026-03-23

**Authors:** Zheng Xia, Huizi He, Zixing Zhou, Shanshan Pan, Sai Zhang

**Affiliations:** Institute of Ultrasonic Testing, Jiangsu University, Zhenjiang 212013, China; xz17768666310@163.com (Z.X.); hehz1010@outlook.com (H.H.); 2222326010@stmail.ujs.edu.cn (Z.Z.); polla_shanshan@163.com (S.P.)

**Keywords:** acoustic imaging, lateral resolution, super-resolution, deep learning, acoustic metamaterials, diffraction limit

## Abstract

Acoustic imaging, especially ultrasound, underpins a wide range of applications from non-destructive evaluation to medical and materials analysis, yet its performance is ultimately constrained by lateral resolution. This review systematically summarizes recent advances in overcoming diffraction-limited resolution, encompassing traditional focusing techniques, transducer optimization, physical metamaterial lenses, and methods based on algorithmic optimization and deep learning technologies. It comprehensively covers approaches for enhancing acoustic lateral resolution, compares the differences and respective advantages and disadvantages of various methods, and proposes clear directions and recommendations for future research. This work provides robust guidance for subsequent research trends and development opportunities in higher-resolution acoustic imaging.

## 1. Introduction

Acoustic imaging technology utilizes the propagation and scattering of sound waves to non-invasively or minimally invasively detect structures and materials, making it widely applied in medical ultrasound examinations, underwater sonar, and ultrasonic non-destructive testing (NDE). High-frequency ultrasound, owing to its non-ionizing nature, real-time capabilities, and relative cost-effectiveness, has seen extensive adoption in clinical and industrial fields. Despite these advantages, spatial resolution remains a critical bottleneck. In most practical acoustic imaging applications, spatial resolution is decomposed into axial (along the beam direction) and lateral (across the distance direction) components. While axial resolution can typically be enhanced by increasing bandwidth (shortening the effective pulse duration), lateral resolution is primarily constrained by the acoustic beam width. Consequently, it faces strong limitations imposed by the diffraction limit [1], finite aperture size, and focusing (i.e., f-number). This results in lateral resolution being depth-dependent, achieving optimal performance only near the focal region while rapidly degrading in defocused areas—a phenomenon particularly constraining for miniature aperture devices. Physically, diffraction effects dictate that lateral resolution scales with the wavelength and the beam’s f-number (the focal length divided by aperture diameter). Traditionally, improving lateral resolution has required two approaches: shortening the wavelength (increasing the center frequency) or enlarging the effective aperture (reducing the f-number). However, both paths introduce fundamental system-level trade-offs. Higher frequencies induce stronger frequency-dependent attenuation, reducing penetration depth and signal-to-noise ratio. Conversely, excessive focusing (low f-number) compresses the lateral main lobe at the expense of depth of field (DOF) and stability across wide imaging ranges. These limitations directly impact critical applications. In intravascular ultrasound (IVUS), transducer aperture is constrained by catheter diameter, and conventional frequency ranges provide lateral resolution of only hundreds of micrometers, which is insufficient to resolve key microstructures like thin fiber caps. In ultrasonic NDE, detecting minute cracks requires lateral resolution comparable to the wavelength. Despite advances like total internal reflection focusing, practical performance remains constrained by defect size and focusing efficiency. Multiple strategies have been proposed to address lateral resolution limitations. For example, the focused IVUS probe manufactured using a mechanically ‘indented’ transducer element demonstrates the effectiveness of micro-scale geometric focusing in enhancing lateral resolution under aperture-limited conditions [2], while ultra-high frequency (UHF) technology faces severe attenuation issues. Coded excitation techniques have been extended to the UHF band, improving the signal-to-noise ratio [3,4]. Synthetic aperture technology enhances the lateral resolution of IVUS and reduces artifacts. Model-driven super-resolution reconstruction often incorporates additional prior or coding mechanisms, transforming the problem into inversion and sparse reconstruction. Examples include compressive sensing super-resolution, random coded interference imaging, PSF modeling, and subspace methods like MUSIC/PC-MUSIC and TR-MUSIC. Furthermore, deep learning-based end-to-end neural network learning methods have accelerated and stabilized such super-resolution imaging, with model generalization being a key focus for future research. Another class of approaches aims to relax diffraction limits by recovering or transmitting evanescent components carrying subwavelength details. Most existing subwavelength imaging methods employ “controlled labels”—subwavelength scattering structures that are deposited near the target that are precisely designed or track spatial distributions to generate evanescent waves. Examples include metamaterials [5,6,7,8,9,10,11,12,13,14,15,16,17,18,19], subwavelength scattering media in time-reversal systems [20,21,22], and contrast agent flow [23,24,25]. Existing reviews often focus on single techniques or specific imaging modalities, lacking systematic classification and comprehensive evaluation of these approaches. This review proposes a new classification framework, grouping these methods into distinct technical categories and comparing their principles and application scenarios to provide readers with a more holistic perspective. It explicitly identifies the gaps addressed and contributions made by this review.

**Figure 1:**
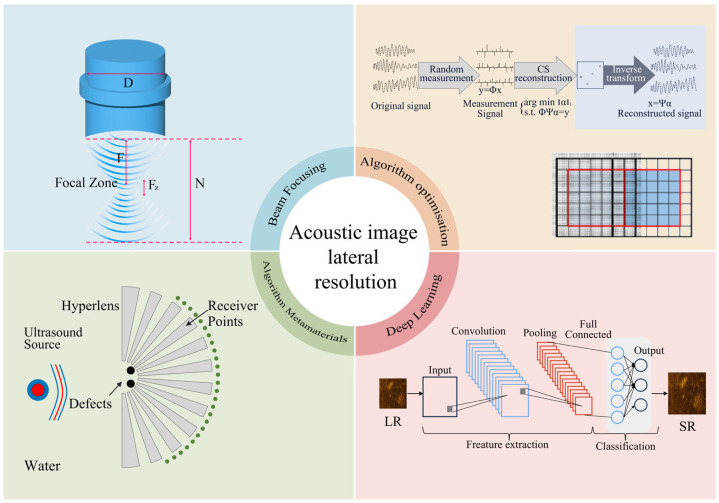
Schematic diagram of technical classifications for improving acoustic image resolution. Adapted from Refs. [26,27]. Reprinted with permission from Ref. [28]. Licensed under CC BY 4.0.

This paper will proceed in the following sequence, as illustrated in Figure 1, which summarizes the method categories discussed in this paper. We will comprehensively elaborate on hardware optimization at the physical layer, followed by super-resolution imaging at the algorithmic level and through deep learning implementation, culminating in subwavelength imaging methods that overcome diffraction limits via acoustic metamaterials.

## 2. Diffraction-Limited Lateral Resolution Enhancement Based on Physical and System-Level Optimization

**Figure 2:**
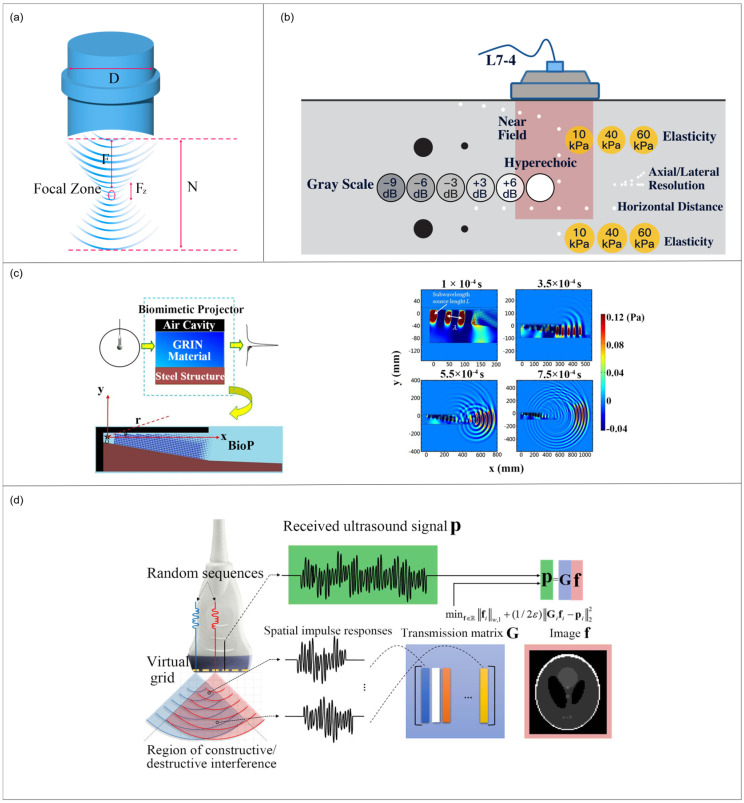
(**a**) Acoustic-focused ultrasound imaging; (**b**) experimental results for CIRS Phantom (Adapted from Ref. [29]); (**c**) **Left** panel shows schematic diagram of the BioP; **right** panel shows simulations of wave propagation produced by the BioP (Reprinted with permission from Ref. [30]. Copyright 2014 AIP Publishing); (**d**) system description of the proposed method (Reprinted with permission from Ref. [31]. Licensed under CC BY 4.0).

As for computational approaches, however, they heavily rely on prior information about the object, such as sparsity, object shape, and edge smoothness. For focusing lenses, the Sparrow criterion is commonly used for theoretical estimation, which states that

(1) $$\delta \approx 1.02\frac{Z_{0}\lambda }{d}$$

where $Z_{0}$ is the focal length, d is the transducer diameter, and λ is the wavelength corresponding to the centre frequency of the transducer. Equation (1) indicates that the lateral resolution improves with a larger aperture (larger d) and a shorter wavelength (higher center frequency), while it degrades as the focal length $Z_{0}$ increases (i.e., a larger f-number $Z_{0}/d$). Figure 2a illustrates a hardware optimization scheme achieving high resolution in acoustic imaging through the use of a focusing lens. The cylindrical lens (diameter D) converges incident sound waves (represented by arc-shaped ripples) into the focal zone, forming a spatially concentrated energy region (F and Fz denote the focal point and focal zone range). This physical convergence constrains the acoustic beam laterally, thereby enhancing the system’s lateral resolution. The parameters F and N on the right further characterize the focal point and acoustic properties, illustrating the fundamental principle of optimizing sound field distribution through lens design to achieve high-resolution imaging. Sparse array designs reduce the channel count while preserving resolution. Nili et al. [29] proposed using two subarrays whose element spacings are coprime multiples; as illustrated in Figure 2b, micro-steering within each subarray and multi-angle compounding are used during beamforming. This synthetic aperture nearly doubles lateral resolution and greatly expands the field of view, even with many fewer active elements. In practice, the coprime sparse array maintained high lateral detail and uniformity despite a drastic channel reduction, demonstrating that clever geometry can sustain subwavelength resolution with simpler hardware.

For example, Zhang et al. [30] developed a dolphin-inspired ‘BioP’, as shown in the transducer in Figure 2c, featuring an internal air cavity, a GRIN lens, and a steel shell. This biomimetic design steers waves into a narrow beam, yielding a main lobe pressure of about three times higher and a beam width that is ten times smaller than a comparable subwavelength source. It thus breaks the directivity limit for miniaturized sources, offering a novel, broadband, and energy-efficient route to subwavelength lateral-resolution improvement.

Innovative transmission schemes have been proposed to break the diffraction limit by creating highly incoherent intonation fields. Ni and Lee [31] proposed an ultrasound imaging method that overcomes the diffraction limit by employing non-focalized randomly encoded wavefronts in place of conventional focalized pulses. All array elements simultaneously emit distinct encoded waveforms, generating complex interference patterns within tissue. Received echoes are reconstructed using ℓ_1_-based compressed sensing and a pre-measured transmission matrix. This approach achieves an ultra-high resolution of approximately 0.25 mm (roughly half the wavelength at 3 MHz), representing a fourfold improvement over conventional DAS imaging. Its principle lies in the unique response of each scatterer to the randomly encoded excitation, enabling sparse reconstruction to recover sub-diffractive details. Additionally, biomimetic projector designs are explored as an alternative hardware strategy to enhance lateral resolution.

A row–column (R–C) matrix array reduces wiring complexity, and Taghavi et al. [32] achieved ~43 µm resolution (~1/6 wavelength) in 3D microvascular imaging, surpassing the diffraction limit and proving subwavelength feasibility. Similarly, Wang et al. [33] use a simple 1D array without elevational focus; it captured ~2.9× more off-plane detail (25.6% → 75.3%) and reached volumetric coverage comparable to a 2D matrix with ~32× fewer elements. Together, these approaches enable high-resolution 3D imaging with greatly reduced hardware demands.

Hardware developments have complemented signal strategies by broadening the bandwidth and reconfiguring array geometry. Cai et al. [34] fabricated a layered transducer that simultaneously generates low- (∼1 MHz) and high-frequency (∼3 MHz) pulses. By temporally overlapping these emissions, the probe produces an ultra-wideband ‘quasi-unipolar’ pulse with a high peak-pressure ratio, boosting the efficiency of super harmonic imaging (i.e., higher harmonics generated in tissue) and improving axial resolution. However, lateral resolution remained limited by the finite aperture. To overcome this, Cai et al. [35] introduced a dual-angle fusion method using a 0.9/3.9 MHz focused probe. They acquired images at a ±45° incidence and coherently fused them, achieving a lateral resolution of ≈0.23 mm (∼0.6 wavelengths) and an axial resolution of ≈0.05 mm (∼0.13 wavelengths) when imaging a custom phantom. This two-angle approach significantly sharpens transverse details beyond what single-angle multi-frequency imaging can achieve.

These studies collectively indicate that the lateral resolution at the diffraction limit is constrained by the coupling trade-off relationship among the physical characteristics of the aperture, the diversity of transmission, and the stability of reconstruction. Lens-based focusing and traditional beam shaping remain the most robust and transferable techniques, but their gain is essentially limited by the number of apertures, attenuation, and depth of field. Sparse/relatively prime arrays and engineered projectors can enhance lateral details by reducing the number of channels or improving directivity; however, their performance highly depends on calibration accuracy, side lobe/ghost control, bandwidth, and manufacturability. Overall, the most scalable approach might be a hybrid solution that combines moderate hardware encoding and physical information reconstruction techniques and undergoes rigorous validation under real conditions.

## 3. Super-Resolution Based on Algorithm Optimization and Deep Learning

### 3.1. Super-Resolution Achieved Through Algorithm

To overcome the diffraction resolution limit in acoustic imaging, research has progressively integrated physical modelling with advanced signal processing. Early methods employed inversion and deconvolution based on the point spread function (PSF) [36,37] of the system, enhancing fidelity but exhibiting sensitivity to modelling accuracy and poor noise robustness. Subsequently developed nonlinear beamforming techniques (such as F-DMAS [38]) enhance contrast and detail resolution through signal multiplication and filtering. Sparse representation and compressed sensing leverage signal sparsity to reconstruct high-resolution images from limited data, suppressing noise through regularization. Subspace methods (e.g., MUSIC) further achieve multi-target resolution below the diffraction limit, though these are constrained by noise and model assumptions. Recent frequency-domain and multi-scale approaches (e.g., FFTR-MUSIC) enhance dense target separation by integrating multi-frequency information and correcting phase. Sharabati [39] proposed a weighted regression method in the field of grey-scale ultrasound imaging to achieve super-resolution imaging. Overall, PSF modelling addresses system blurring, F-DMAS refines beamforming, sparse reconstruction overcomes data limitations, subspace methods transcend classical resolution limits, and frequency-domain fusion restores coherence under bandwidth constraints. However, each individual technique remains constrained by practical challenges, including noise, insufficient prior information, and hardware bandwidth limitations. Consequently, hybrid strategies integrating multiple approaches represent a pivotal direction for advancing lateral super-resolution imaging.

#### 3.1.1. Lateral Super-Resolution Imaging Methods Based on PSF Inversion

Liu and Lu [40,41] both conducted research on PSF algorithms. Liu investigated the impact of PSF on the super-resolution imaging performance of ultrasound images, particularly in scenarios requiring accelerated data acquisition. The results indicated that the Balanced SOFI (BSOFI) method could be employed in SR-US (super-resolution ultrasound) to achieve superior imaging performance. Lu, meanwhile, proposed a PSF-weighted super-resolution imaging method based on PSF modulation techniques. This approach is more readily implementable. It advances techniques for 4D super-resolution imaging of passive targets or wave sources.

In summary, algorithm-driven super-resolution techniques such as PSF modeling, sparse reconstruction, and subspace-based approaches have effectively extended resolution boundaries without hardware modifications. However, their reliance on accurate priors and sensitivity to noise pose practical challenges.

#### 3.1.2. Compressed Sensing and Sparse Representation Driving Super-Resolution Imaging

**Figure 3:**
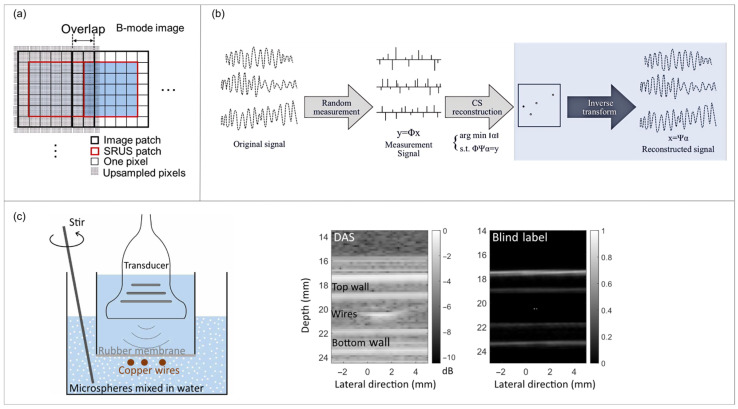
(**a**) For a compressed sensing-based reconstruction of the SRUS image, upsampled image patches (thick black square) were used by overlapping two pixels between adjacent image patches to remove false localization of point targets near boundaries and to correctly build SRUS patches (red thick square). By stitching SRUS patches side by side, a complete SRUS image was acquired from a B-mode image (Reprinted with permission from Ref. [26]. Licensed under CC BY 4.0). (**b**) Reconstruction signal diagram (Adapted from Ref. [27]). (**c**) **Left** panel shows a schematic of the experiment setup; **right** panel shows reconstructed B-mode images obtained using delay-and-sum (DAS) and blind-label imaging system (Reprinted with permission from Ref. [45]. Licensed under CC BY 4.0).

In recent years, compressed sensing (CS) theory has been extensively studied in acoustic imaging fields such as ultrasound. Its principle primarily serves to reduce sampling volume and enhance imaging speed. Some research indicates that through the design of ingenious undersampling and reconstruction methods, the CS framework can also directly improve a system’s spatial resolution, particularly its lateral resolution. For instance, Anand et al. [42] employed a Gaussian-distributed receiver array under sampling and utilized the Wave atom transform as a sparse basis for compressed reconstruction of conventional focused beam imaging. Experimental results demonstrate that this CS approach nearly doubles the lateral resolution compared to conventional methods. In ultrasound localization microscopy, CS algorithms enable resolution of closer-spaced targets; the principle is illustrated in Figure 3a. Studies indicate that the ℓ_1_-homotopy (L1H-CS) algorithm reduces the minimum resolvable distance between two points, from 96.3 μm (lateral) to 27.5 μm [26]. This method significantly enhances localization accuracy through block overlap but increases computational complexity in stitching and reconstruction. The iterative algorithmic computation process is resource-intensive, and real-time performance requires improvement. Future approaches may include GPU acceleration or deep learning algorithm optimization to accelerate reconstruction speed. Additionally, extending this method to three-dimensional ultrasound imaging or studying dynamic moving targets warrants further investigation. More recently, ‘blind labelling’ super-resolution imaging techniques have been developed. By introducing random particles into the imaging region, information below the diffraction limit can be encoded and reconstructed. Consequently, numerous scholars have conducted research centered on CS algorithms in recent years.

Gifani et al. [43] introduced sparse reconstruction methods into ultrasound temporal domain super-resolution imaging, employing Bayesian compressive sensing models to achieve high-resolution reconstruction of high-frame-rate dynamic sequences. Wang [27] subsequently proposed an orthogonal sparse dictionary based on linear frequency modulation, enabling high-quality reconstruction at low sampling rates while significantly enhancing the signal-to-noise ratio. This method leverages the sparse characteristics of the frequency domain to accelerate the reconstruction process (Figure 3b), offering practicality and good compatibility, thereby providing algorithmic support for future intelligent ultrasound diagnosis. However, this method relies on precisely known pulse signal models and is sensitive to waveform variations or system parameters; dictionary pre-training requires calibration and offers limited flexibility. Adaptive or online learning dictionaries represent a promising research topic and direction for future exploration. In addition, Song et al. [44] combined sparse dictionary learning with marginal information, employing an adaptive overcomplete dictionary and marginal insertion strategy to achieve superior performance over conventional methods in both subjective visual assessments and objective metrics. Collectively, these studies demonstrate the pivotal role of sparse priors in enhancing ultrasound image quality, amplifying detail representation, and suppressing noise.

Lin and Ma Chu [45] proposed a ‘blind label’ subwavelength ultrasound imaging’ method (Figure 3c), aiming to overcome the resolution limitations imposed by the diffraction limit in acoustic imaging. This method preserves high-frequency information by introducing random particles (‘blind tags’) that require no precise control, thereby converting the target’s evanescent waves into propagating waves. Using compressed sensing algorithms to reconstruct images from randomly scattered far-field data, experiments achieved a lateral resolution of 0.24 wavelengths (more than a tenfold improvement over conventional methods), significantly enhancing applicability in complex real-world environments (Figure 3c). This method relies on introducing random scatterers into the imaging region and assumes a known PSF. It is sensitive to particle size, concentration, and distribution. Non-uniform attenuation and motion in real tissue further compromise reconstruction stability and introduce higher complexity. Future work should explore in vivo “blind labeling” using biocompatible contrast agents, combined with joint optimization of scatterer parameters and motion correction, to achieve a more robust trade-off between resolution and signal-to-noise ratio.

#### 3.1.3. Subspace-Based Super-Resolution Acoustic Imaging via MUSIC and Frequency-Focused TR-MUSIC and FFTR-MUSIC Algorithms

The MUSIC (Multiple Signal Classification) algorithm is a classic signal processing technique that is widely used in array signal processing, direction finding, and frequency estimation. Its main goal is to extract the characteristics of signals by analyzing the covariance matrix of received signals, which helps in determining the position or frequency of the signal sources. The MUSIC algorithm works by separating the signal subspace from the noise subspace to estimate the direction or frequency of signals. Zheng et al. [46] provide a detailed description of the advantages of this algorithm in signal source separation. By performing feature space analysis of echo signals using the MUSIC algorithm, precise localization of sound sources can be achieved to a significant extent, thereby significantly improving the lateral and depth resolution of imaging. This method is particularly suitable for multi-target imaging and clinical scenarios with extremely high requirements for detail resolution. Although traditional methods like TR-MUSIC perform well in multi-target sound source localization, they struggle in noisy environments. To address this, Foroozan and Sadeghi et al. [47] introduced FFTR-MUSIC, which combines frequency focusing with TR-MUSIC. This method concentrates multi-frequency domain information into a “super-resolution” space using a frequency-tuned measurement matrix to correct phase differences, improving the resolution for sub-wavelength targets. Simulations show that FFTR-MUSIC successfully resolved two targets separated by λ/2, unlike TR-MUSIC, which failed due to insufficient coherence. This demonstrates the effectiveness of combining frequency reconstruction with subspace techniques for enhancing lateral resolution.

**Figure 4:**
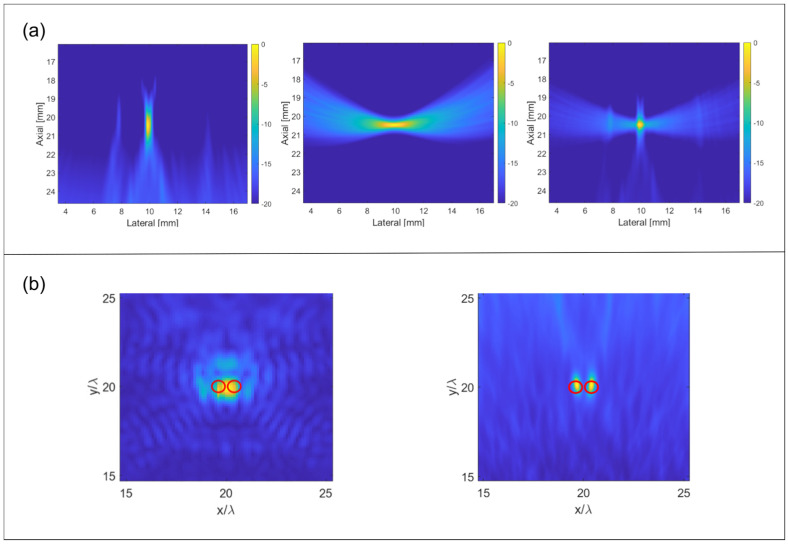
(**a**) B-mode images of TR-MUSIC, R-MUSIC imaging technique, and proposed method (Reprinted with permission from Ref. [48]. Licensed under CC BY 4.0). (**b**) Imaging results and corresponding indicator profile from experimental data with two damages separated by 10 mm (Reprinted with permission from Ref. [49]. Licensed under CC BY 4.0).

Concurrently, the R-MUSIC (Range-MUSIC) method was also proposed. By analyzing the temporal domain characteristics of echo signals and exploiting phase rotation rate differences across distinct frequency sub-bands, R-MUSIC effectively achieves axial super-resolution without enhancing lateral resolution. To simultaneously enhance both lateral and axial resolution, Zheng et al. [48] combined TR-MUSIC with R-MUSIC, proposing a novel super-resolution imaging method. This hybrid approach preserves TR-MUSIC’s favorable lateral resolution while achieving super-resolution in the axial direction. Experimental and simulation results demonstrate that the combined method outperforms either TR-MUSIC or R-MUSIC when employed individually. Figure 4a compares the B-mode imaging results obtained using TR-MUSIC alone, R-MUSIC alone, and their fusion. The results demonstrate that the fusion method inherits the high lateral resolution characteristic of TR-MUSIC while also possessing the super-resolution capability of R-MUSIC in the axial (depth) direction. In other words, this hybrid approach significantly enhances the detection capability of minute defects while preserving the original imaging advantages. Meanwhile, Xu et al. [49] compared damage imaging methods based on ultrasonic-guided waves, focusing particularly on the time-domain DAS (Delay-and-Sum) method and the DORT-MUSIC method by incorporating the MUSIC algorithm. The study demonstrated that the DORT-MUSIC method, by integrating the time-domain inversion operator (TRO) with multi-signal classification (MUSIC), significantly enhanced imaging resolution. Figure 4b presents the imaging results and metric curves for two 10 mm spaced cracks in the laboratory. The comparison between frequency-domain DAS and DORT-MUSIC further validates the DORT-MUSIC method’s superiority in identifying minute defects. Although the illustrated results support the method’s effectiveness, note that the current findings were obtained under specific experimental conditions. Parameter selection (e.g., fusion coefficients z1, z2) significantly influences outcomes, and the analysis is limited to the 10 mm spacing scenario. Future work should extend to different spacings, noise levels, and material structures to comprehensively evaluate the method’s robustness and practicality.

#### 3.1.4. Comparison and Analysis Summary

In conclusion, the super-resolution improvements described in this chapter are mainly achieved through PSF, compressive sensing, and the use of subspace separation. These studies collectively indicate that resolution enhancement is not an automatic process. The best results are achieved when the method assumptions hold true, namely, propagation and phase stability, sufficient signal-to-noise ratio, and a sufficient number of snapshots to reliably estimate the covariance, with limited model bias. Once these conditions are relaxed (such as spatially varying point spread functions/aberrations, motion/non-stationarity, coherent multipath or correlated noise), the mechanisms for sharpening point targets may also amplify artifacts or produce false peaks, thereby leading to an overinterpretation of visual sharpness as real structure. Therefore, the more convincing conclusions in the existing evidence are conditional. Subspace methods tend to significantly enhance separability when combined with model order testing; while sparsity/CS pipelines can achieve excellent results on controlled data, they need to be carefully handled with boundary conditions and robustness verified before being widely applied.

### 3.2. Deep Learning Achieves Super-Resolution Imaging

Deep learning has emerged as an effective approach for enhancing the lateral resolution of acoustic images. Existing super-resolution methods mainly include CNN-based reconstruction, GAN-based adversarial learning, and Transformer-based modeling. These methods achieve superior lateral resolution enhancement and detail recovery compared with traditional techniques, although challenges remain in data dependence and generalization. This paper primarily reviews the recent progress made in CNN and GAN-based lateral super-resolution, with a brief discussion of emerging Transformer-based approaches.

#### 3.2.1. CNN-Type Methods and Improved CNN Models

CNN-based super-resolution methods have become a dominant approach for enhancing the lateral resolution of acoustic images. These techniques establish an end-to-end mapping from low to high resolution by progressively extracting and reconstructing local details through layered convolutions. A summary of representative CNN-based super-resolution methods for improving lateral resolution in acoustic imaging is provided in Table 1. Early applications demonstrated their core strength in capturing fine structural details with high fidelity. For instance, Deep-ULM represents a pioneering deep-learning-based method for ultrasound localization microscopy, utilizing a fully convolutional U-Net to achieve real-time ultra-high-resolution vascular imaging from high-density microbubble data [50,51,52,53]. Similarly, in scanning acoustic microscopy (SAM), Makra et al. [54] successfully mapped low-frequency (180 MHz) images to high-frequency (316 MHz) references using deep learning, significantly approximating the lateral resolution of true high-frequency systems. This foundational work was extended to other imaging modalities, such as the IDNet proposed by Lu et al. [55] for cardiovascular imaging, which employed multi-scale Inception modules to match the quality of multi-transmission composites with drastically fewer transmissions.

Subsequent research has focused on architectural refinement and integration with physical models to further push performance boundaries. In plane-wave imaging, Perdios et al. [56]. first employed a U-Net to map single-frame data to synthetic aperture quality, reporting over 25% lateral resolution improvement. They subsequently advanced this in 2022 with a two-stage CNN that first creates a preliminary image via back projection before using a residual network to remove artefacts, achieving quality comparable to extensive frame compounding [57]. Parallel innovations in beamforming were introduced by Nguon et al. [58], who designed an end-to-end deep beamforming network based on an enhanced U-Net, yielding substantial gains in PSNR and CNR while reducing image width metrics.

To further enhance detail reconstruction, recent models have incorporated sophisticated mechanisms like multi-scale processing and attention. Kim et al. [59] combined a Multi-Scale Deep Encoder-Decoder Network (MSDEPC) with phase consistency to accentuate edges and improve spatial resolution. Following this direction, Lei et al. [60] developed the Attention Convolutional Neural Network (AGCNN), integrating attention gates and skip connections for multi-scale feature fusion, which delivered superior PSNR and SSIM on tissue data. Conversely, other studies have designed specialized networks for specific challenges. Long et al. [61] proposed the SRSS-Net for sparse-array shear-wave imaging, which synergizes a physical refraction wave algorithm with a CNN enhancer to suppress artefacts and accelerate computation, albeit with a noted dependency on extensive training data. Tamang and Kim [62] addressed general ultrasound super-resolution with their Symmetric Serial CNN (SS-CNN), a unique dual-branch architecture that effectively preserves underlying texture, outperforming other learning-based methods on public benchmarks.

Beyond entirely new architectures, refinements to established networks have also proven highly effective. A notable example is the work of Wang et al. [63], who enhanced the classic VDSR network for SAM images by introducing a bottleneck structure, replacing ReLU with Leaky ReLU to preserve negative-value information, and employing a clipped ReLU in the output layer. This optimized model not only achieved a higher PSNR and SSIM but also boosted the accuracy of a downstream CNN defect classifier to 97.9%, demonstrating the practical impact of superior reconstruction.

**Table 1:** Architecture and illustrative diagrams of CNN and their enhanced models for acoustic super-resolution imaging.

	Perdios et al. [57]	Nguon et al. [58]	Tamang & Kim [62]	Lei et al. [60]	Wang et al. [63]	Makra et al. [64]
**Model Architecture**	CNN-based image reconstruction	Modified U-Net	Symmetric Series CNN (SS-CNN)	Enhanced CNN with attention mechanism	Deep CNN (IDNet)	Deep Learning (DL)
**Image Type/Resolution**	Single plane-wave ultrasound	Plane-wave ultrasound	Ultrasound medical images	Low-frequency ultrasound	Diverging-wave ultrasound	Scanning acoustic microscopy (SAM)
**Performance Metrics**	PSNR, CNR, LR	LR improved by 29.6%	PSNR, SSIM	PSNR, SSIM	CNR, LR, CR	NRMSE, PSNR
**Image Quality Improvement**	Image quality comparable to gold-standard synthetic aperture imaging	Improved lateral resolution by 29.6%	Improved resolution and textural quality	Improved resolution, suppressed noise	Image quality equivalent to 31 diverging waves from 3 waves	Enhanced 180 MHz SAM image resolution
**Applications**	Functional ultrasound neuroimaging	Cardiovascular imaging	General medical diagnostics	Breast cancer early detection	Cardiovascular imaging	Biological tissue imaging

In summary, CNN-based methods have substantially advanced the field of acoustic image super-resolution through continuous architectural innovation—from foundational U-Net applications to the integration of multi-scale, attention, and physics-informed modules. These approaches consistently outperform traditional algorithms in detail fidelity and noise suppression. However, their performance often relies heavily on substantial high-quality training data, and generalization in complex real-world scenarios remains a challenge. Current research efforts are therefore increasingly focused on reducing this data dependency and improving model robustness to facilitate broader clinical and industrial applications.

#### 3.2.2. GAN-Type Methods and Improved GAN Models

GANs introduce adversarial loss through the competitive mechanism between discriminators and generators, enabling the production of high-resolution images with more authentic details. Compared to simple pixel reconstruction, GANs more effectively restore ultrasound image details (such as suppressing speckle noise), making super-resolution reconstructions closer to genuine high-resolution images in terms of perceived quality and texture authenticity. Existing research encompasses supervised conditional GAN frameworks (mapping low-quality data to high-quality images) and unsupervised CycleGAN variants (ensuring reconstruction quality through cyclic consistency constraints when high-resolution ground truth data are scarce). However, GAN training is complex, requiring meticulous loss-function design and balancing training stability.

Within supervised super-resolution applications, researchers have leveraged GANs to enhance ultrasound image quality. Mishra et al. [64] applied GANs to remove speckle noise from ultrasound images, proposing a structure-oriented adversarial model. Their generator incorporated residual connections and fused adversarial loss with structural fidelity loss, thereby reducing noise while preserving tissue structural features. Nair et al. [65] designed a GAN model inputting single-frame plane-wave RF data, with the generator outputting synthetic aperture B-mode ultrasound images and corresponding segmented images. Through extensive adversarial training on simulated data, it achieved a PSNR of approximately 29.4 dB in simulation tests. Wang et al. [66] proposed a conditional GAN-based super-resolution reconstruction, mapping single-pass plane-wave RF data to B-mode ultrasound images. Their output images exhibited significantly higher signal-to-noise ratios than traditional delay-summing algorithms, with the correlation coefficient between reconstructed images and 75-frame composite imaging results improving from 0.641 to 0.976.

Furthermore, addressing the trade-off between resolution and penetration depth in ultrasound imaging, researchers proposed enhanced GAN architecture. Goudarzi et al. [67] constructed a GAN framework employing a compact U-Net as the generator. Inputting low-frequency transmit RF signals and their envelope diagrams enabled the generation of high-frequency ultrasound images that substantially enhanced axial resolution while maintaining penetration depth. Khor et al. [68] introduced a wavelet reconstruction module into the generator within the WGAN framework, employing a band-wise normalization strategy to preserve high-frequency image details whilst reducing noise.

Beyond supervised models, unsupervised super-resolution methods based on Cy-cleGAN have also advanced in recent years, enabling high-quality reconstruction when high-resolution training samples are scarce. Liu et al. [69] pioneered a self-supervised ultrasound image super-resolution framework using CycleGAN trained solely on low-resolution images. This approach employs a cycle consistency loss to constrain the generator and discriminator, ensuring that reconstructed images maintain consistency with the original input after downsampling. Ding et al. [70] proposed a two-stage zero-sharpening super-resolution CycleGAN, integrating zero-sharpening super-resolution with CycleGAN. They incorporated residual modules and attention mechanisms into the generator, using a cycle consistency loss to jointly constrain the LR → SR → LR and HR → LR → SR processes. Si et al. [71] designed a residual attention dual-discriminator CycleGAN for plane-wave imaging. This achieves unsupervised high-quality reconstruction through residual attention modules and dual discriminators in both time and frequency domains, yielding approximately 7.8% and 22.2% improvements in PSNR and SSIM, respectively, compared to a baseline CycleGAN.

Table 2 summarizes key GAN-based and enhanced GAN frameworks for acoustic super-resolution imaging, emphasizing how adversarial learning enhances perceptual sharpness and speckle/texture restoration over traditional pixel-wise CNN methods. Across studies, conditional GAN variants primarily sharpen vessel/tissue boundaries and boost visual realism, while CycleGAN-inspired or weakly supervised approaches reduce reliance on paired high-resolution (HR) and low-resolution (LR) training data. However, these benefits carry significant risks: GANs can generate visually plausible but clinically or physically inaccurate structures, training is often unstable and sensitive to loss weighting, and perceptual gains may not align with task-specific fidelity or quantitative resolution metrics. Future efforts should focus on incorporating physics- and beamforming-aware constraints, uncertainty quantification to detect hallucinations, and rigorous cross-domain validation, including scanner/protocol generalization, with standardized benchmarks and clinical reader studies to ensure that super-resolution advancements yield reliable diagnostic utility.

#### 3.2.3. Transformer Methods

**Figure 5:**
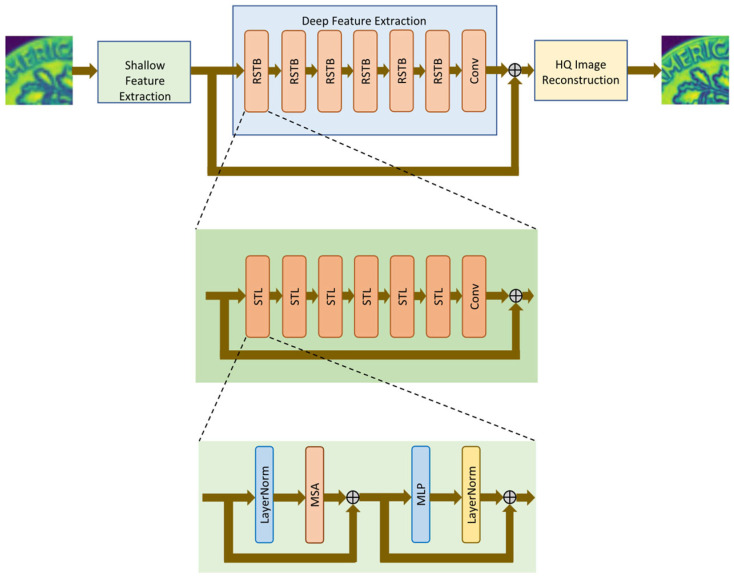
This illustration demonstrates the high-resolution image inpainting strategy for SAM images (Reprinted with permission from Ref. [74]. Licensed under CC BY 4.0).

In recent years, Transformer models have been introduced into the field of super-resolution, owing to their global modelling capabilities. Cheng et al. [72] proposed the MSCT model, which combines multi-scale adaptive convolutions with hybrid Transformer modules for super-resolution reconstruction of medical images (CT, MRI). On public datasets, it achieved an average PSNR of approximately 21.96 dB and an SSIM of approximately 0.8053, outperforming most traditional methods. In acoustic microscopy imaging, Sharma et al. [73] proposed the HDL-SAM hybrid network, integrating the Transformer-based SwinIR with super-resolution restoration techniques to achieve 4× magnification. The reconstructed results attained approximately 0.92 SSIM and 31.60 PSNR, significantly outperforming single-model approaches. Comparative studies by Banerjee et al. [74] further demonstrate that Transformer models (such as SwinIR) excel in SAM image super-resolution tasks, with the principle and effect illustrated in Figure 5, achieving an average SSIM of approximately 0.95 and PSNR of approximately 35. Somani et al. [75] proposed a hypergraph convolution-based super-resolution method for SAM images. By constructing hypergraphs to mine high-order correlations between the target region and background, they employed a two-stage generative adversarial network for restoration. This approach achieved advanced performance in 4× super-resolution tasks with a SSIM = 0.82 and PSNR = 27.96, outperforming mainstream deep learning approaches such as AOTGAN and DeepFill v2. It also demonstrates robust noise resistance. However, its high parameter count and computational cost may hinder real-time applications.

Three distinct conclusions emerge across CNN-, GAN-, and Transformer-based models. First, supervised CNN algorithms often deliver the most reliable numerical improvements under matched acquisition conditions, but they typically trade off clarity for smoothness and remain susceptible to domain shifts when probe, frequency, or beamforming settings change. Second, GAN-based super-resolution techniques are highly effective at restoring texture and boundaries with perceptual clarity, but they carry the greatest risk of ‘plausible errors’; without constraints on perceptual loss, they may generate structures that are inconsistent with measurements, posing particular challenges for quantitative diagnostics. Third, Transformer-based models show promise in reconstructing globally consistent images, but their computational and memory demands are substantial. Unless combined with lightweight designs, this often limits their real-time applicability. To further strengthen the evidence’s persuasiveness, future research should adopt cross-domain validation methods to detect scenarios where image reconstruction outputs may be unreliable. These steps will translate evidence into transferable insights that are applicable to practical applications.

## 4. Super-Resolution Based on Acoustic Metamaterials

By amplifying evanescent waves that would otherwise decay, acoustic metamaterials [12,76] can overcome the diffraction limit and enable super-resolution imaging. Recent advances have dramatically extended this capability, and lateral resolution has been pushed from around the classical diffraction limit down to below λ/100, while imaging has been extended from the near field into the far field. It is important to emphasize that the extremely high subwavelength resolutions reported in the literature (e.g., reaching the order of λ/100) are typically achieved under near-field or quasi-near-field imaging geometries; the imaging target often needs to be positioned close to or even in contact with the lens/metamaterial structure to ensure effective coupling of the evanescent wave. Simultaneously, the image readout plane is often positioned extremely close to the output surface (e.g., at the 0.01λ scale) or even within the structure itself (e.g., sampling at the midplane z=h/2) to capture the amplified evanescent wave component. In the study of Chen [77], an experimental resolution of ‘<λ/100’ in the 2–3 kHz airborne acoustic frequency band is reported, and it explicitly specifies the operating bandwidth (2250–2900 Hz) and the −6 dB bandwidth of the pulse excitation (center frequency ~2580 Hz, −6 dB bandwidth ~650 Hz). Comparison results indicate that even at a reference plane of just 0.01λ away from the output surface, deep subwavelength details may rapidly decay and become difficult to resolve, whereas sampling within the lens significantly enhances resolution capability. For classical aperture-array metamaterial superlenses [10,78,79,80], although imaging at approximately λ/50 is achievable under resonant conditions, they similarly exhibit strong near-field dependence: supplementary experiments demonstrate that images rapidly degrade to indistinguishable levels when the output scanning plane increases from approximately 0.02λ to 0.06λ, reflecting the rapid decay of deep subwavelength information in free space. Therefore, when citing λ/100-level conclusions in reviews, the applicable conditions (near/far-field geometry, operating frequency band and bandwidth definition, measurement/reconstruction aperture, scanning distance/sampling plane position, etc.) should be explicitly stated to avoid confusion with far-field imaging scenarios. These breakthroughs rely on tailored metamaterial lens designs. For example, superlenses exploit negative refraction or localized resonances to recover fine subwavelength details, whereas anisotropic acoustic hyperlenses [9,81] convert evanescent near-field waves into propagating far-field waves. More recently, metasurface-based [82,83,84,85] lens designs have been developed that employ precise subwavelength wavefront control to achieve ultra-high-resolution focusing across a broad frequency band.

### 4.1. Superlens

**Figure 6:**
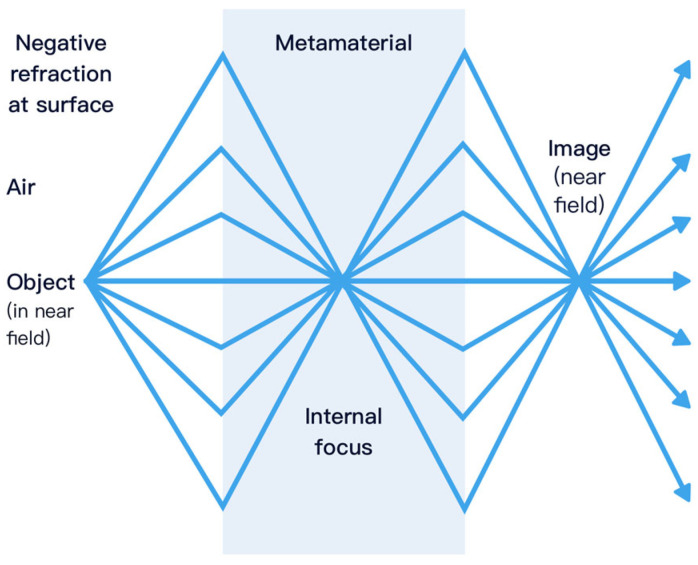
Concept of superlens.

A superlens is a flat slab of negative-index metamaterial that refracts light to the ‘negative’ side at each interface, as shown in Figure 6, causing rays from a near-field object to converge to an internal focus and then re-form an image outside the slab. Unlike conventional lenses, the metamaterial simultaneously refocuses propagating waves and restores evanescent components that carry subwavelength spatial information. By compensating for the natural decay of these near-field evanescent waves, the slab reconstructs a high-fidelity image with features beyond the diffraction limit, yielding true subwavelength resolution in the near field.

**Figure 7:**
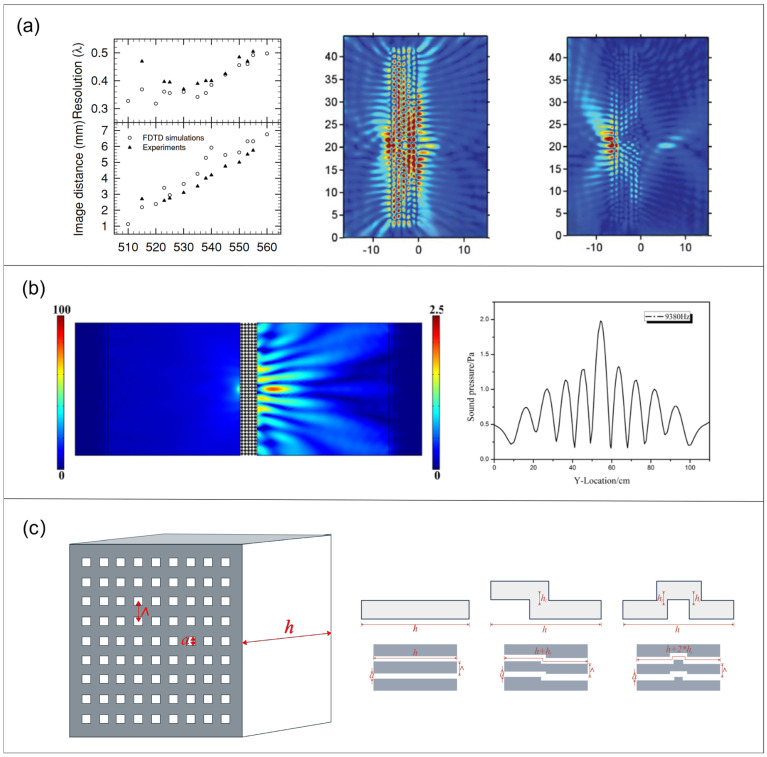
(**a**) Effects of the operating frequency. Lateral resolution and distance of the image as a function of the operating frequency. Results from experiments (Reprinted with permission from Ref. [86]. Copyright 2011 APS Pushing). (**b**) Pressure-field distribution obtained in a simulation of focusing with a point wave source at a frequency of 9380 Hz and pressure intensity of the focusing spot (i ≈ 13.4 cm) behind the lens (Reprinted with permission from Ref. [90]. Licensed under CC BY 4.0.). (**c**) Schematic diagram of AML and schematic diagram of structure and effective length of holes (Adapted from [91]).

Planar metamaterials and film/Helmholtz-cavity structures use negative refraction or localized resonance for subwavelength focusing; Fano-type inter-unit coupling and multiple resonance channels broaden the bandwidth, and external-field control (e.g., magnetostriction) enables stable multi-frequency imaging, shifting from a single-frequency to broadband/tunable operation. In 2011, Robillard et al. [86] designed a triangular-lattice steel-pillar photonic-crystal superlens (methanol) that imaged water-borne point sources with ~0.34λ focal spots; the experimental results for its lateral resolution and pressure field at different frequencies are shown in Figure 7a, far below half-wavelength. Addouche et al. [87] extended this to surface waves via a periodic column array, reaching an ~λ/3 resolution with an effective negative index of ≈−1.

Liu et al. [88] proposed localized-resonant metasurfaces with anisotropic effective mass; when the thickness equals integer half-wavelengths, Brillouin waves induce strong spatial modulation and complete evanescent-wave transmission. Infinite equivalent density parallel to the interface drives near-infinite phase velocity, converting evanescent to propagating waves with uniform spatial-frequency amplification (full-wave simulations confirm sub-diffraction imaging).

Kaina et al. [89] broke the symmetry of subwavelength periodic Helmholtz-resonator media to realize dual-negative index and left-handed propagation; a honeycomb array produced a negative band near 420 Hz and a planar lens whose transmitted focal spot was ~1/4 of the lensless case, and resolved two opposite-phase sources separated by ~λ/7. Concurrently, Chen et al. [90] proposed a single-phase acoustic metasurface based on a star-shaped lattice structure that was designed to overcome the diffraction limit and effectively focus sound waves. Utilizing a star-shaped metamaterial constructed from steel, which exhibits a unique negative refractive index and resonance properties, a lightweight and structurally simple acoustic metasurface was engineered that was suitable for aquatic environments. Simulation results demonstrate that this superlens achieves a spatial resolution of 0.39λ at 9380 Hz, surpassing the conventional 0.5λ diffraction limit. The simulated pressure field distribution and focal intensity distribution (Figure 7b) clearly reveal the focusing effect. This research offers a novel solution for super-resolution acoustic imaging, exhibiting significant practical application potential. Yang et al. [91] used strong inter-resonator Fano coupling in a planar HR network to achieve a continuous super-resolution across 570–650 Hz. Liu et al. [92] built a magnetically tunable membrane super-resolution lens (≈6%λ thick), achieving 350–700 Hz operation by modulating tension to shift zero-mass resonance

Loheshwaran Chandran et al. [93] showed in NDT simulations that defect-scattered waves degrade amplitude and add artefacts, depending on the defect position and receive-surface distance—guiding algorithmic compensation and lens optimization. Shi et al. [94] introduced multi-length subwavelength channels forming supercells that support multiple Fabry–Pérot modes at a constant thickness, broadening the super-resolution bandwidth without sacrificing resolution (validated by simulation and ultrasonic imaging). Figure 7c displays the 20.8 kHz pressure field output from this design, alongside the SC-4 normalized results.

### 4.2. Hyperlens

**Figure 8:**
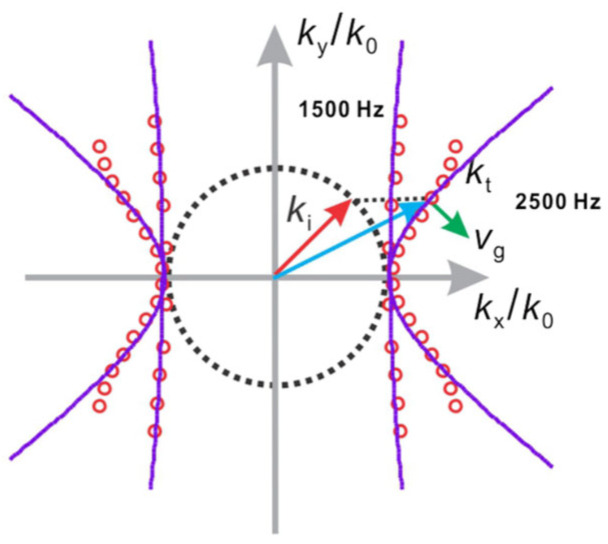
Equal frequency contour (EFC) at two frequencies (Reprinted with permission from Ref. [11]. Copyright 2015 by the American Physical Society).

The hyperlens principle relies on the hyper-surface dispersion of acoustic hyper-surface materials (AHMM), which achieve effective density differences in the x- and y-directions through thin plate arrays. When frequencies fall below a cutoff, the density in the y-direction becomes negative, inducing hyper-surface dispersion. This causes negative refraction and partial focusing of sound waves, enabling the transmission of subwavelength information for subwavelength imaging. In experiments, AHMM demonstrates partial focusing across a broad frequency range and shows subwavelength imaging at specific frequencies. Figure 8 below illustrates the equal-frequency contours (EFC) at two frequencies, showing how the EFC adopts a hyperbolic shape at low frequencies. The solid lines represent EFCs calculated using a lumped model, while the circled markers show results from numerical simulations, thereby confirming the material’s hypersurface dispersion and focusing ability at low frequencies [11].

Acoustic hyperlenses are metamaterial devices that are designed to overcome the diffraction limit in sound imaging by capturing and magnifying evanescent (subwavelength) acoustic waves into the far field. Drawing inspiration from electromagnetic hyperlenses, an acoustic hyperlens uses anisotropic media with unusual dispersion properties to channel high-spatial-frequency information that would normally decay near the source out to a detectable far-field signal.

**Figure 9:**
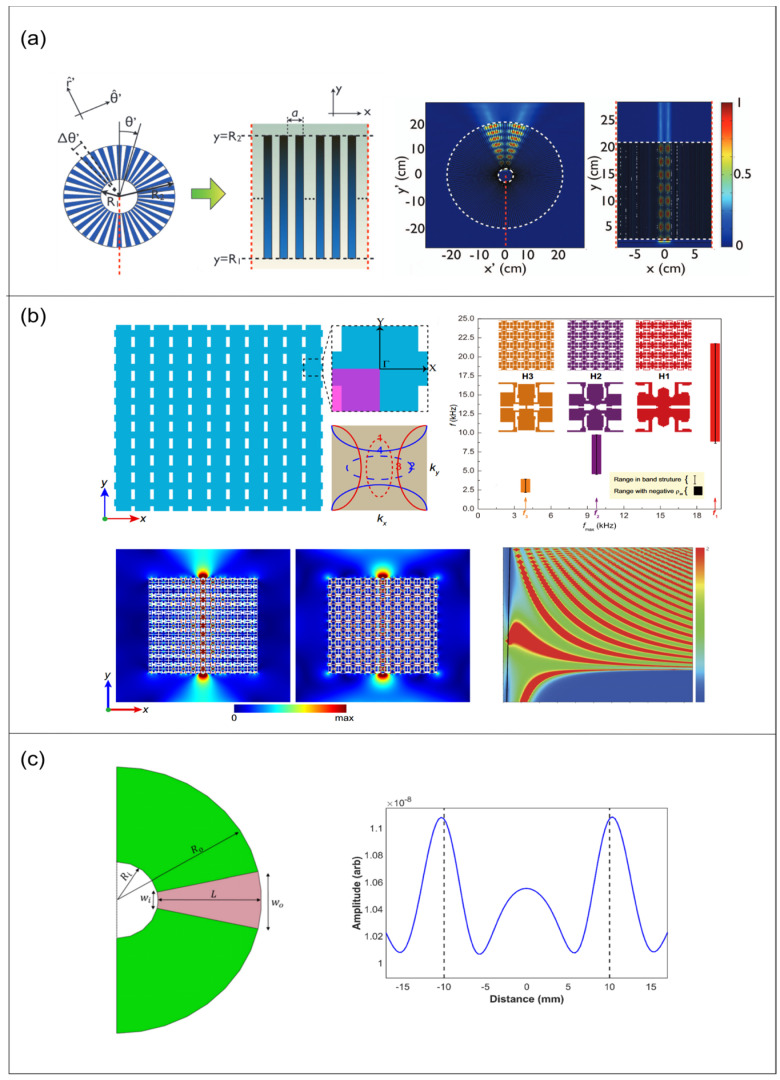
(**a**) **Left** panel is the microstructure of the cylindrical hyperlens; **right** panel shows the pressure intensity pattern at frequency 6.6 kHz of the cylindrical hyperlens (Reprinted with permission from Ref. [95]. Licensed under CC BY 3.0). (**b**) Schematic illustration of an anisotropic metamaterial and the topology-optimized results (Reprinted with permission from Ref. [98]. Licensed under CC BY 4.0). (**c**) Schematic illustration of the geometric parameters involved in the design of cylindrical hyperlens (Reprinted with permission from Ref. [28]. Licensed under CC BY 4.0).

Following the proposal of the acoustic metamaterial lens concept, Li et al. [9] were the first to experimentally realize a cylindrical metamaterial lens (4.2–7 kHz) based on a non-resonant metal–air layered structure. This lens could resolve sound sources separated by 1.2 cm (approximately 1/6λ) and achieve broadband amplification imaging with low loss. However, this structure was only applicable to curved one-dimensional targets [8]. Subsequently, Liang and Li [95] theoretically compared non-resonant anisotropic hyperlenses with localized resonance-based hyperlenses. Figure 9a illustrates the pressure distribution across two lenses at 6.6 kHz; the former enables broadband subwavelength imaging but suffers resolution limitations imposed by geometric magnification; the latter achieves higher resolution (up to λ/50) but operates within an extremely narrow frequency band.

To overcome the geometric constraints of cylindrical structures, Gu et al. [96] proposed the first planar acoustic metamaterial lens based on anisotropic near-zero density (ADNZ) metamaterials. This utilized film-type metamaterials, achieving near-zero density around 976 Hz to transmit high-wavevector components. This device experimentally resolved line sources that were spaced at 0.26λ, restoring details at approximately λ/11, though it remained constrained by narrowband characteristics due to localized resonance.

Regarding dimensional expansion, Hu et al. [97] realized the first three-dimensional acoustic hyperlens. By employing non-resonant anisotropic units on a spherical shell structure, it effectively converted near-field high-wavevector information into propagating waves, achieving broadband far-field super-resolution imaging at 4.6–9 kHz. Their experimental resolution reached approximately λ/8 with about 4.5-fold magnification, enabling reconstruction of complex letter-shaped targets. Extending the pursuit of higher resolution, as shown in Figure 9b, Dong et al. [98] utilized topology optimization to design broadband single-phase hyperbolic elastic metamaterials, which achieved a record imaging resolution of ~λ/64 for longitudinal waves at ultra-low frequencies. This demonstrates the significant potential of inverse-design strategies in pushing the limits of super-resolution imaging within elastic systems. Additionally, this novel cylindrical metamaterial lens structure for ultrasonic imaging, proposed by Ali and Rajagopal [28], employs anisotropic metamaterials to convert evanescent waves into propagating waves, thereby achieving far-field magnification imaging of subwavelength features. This approach experimentally achieved 5× super-resolution imaging of defects with spacings of approximately λ/3 in the ultrasonic frequency band, demonstrating its potential applications in non-destructive testing and biomedical imaging. Figure 9c presents the numerical results for the cylindrical superlens structure and the 10× magnification planar superlens.

### 4.3. Metalens

**Figure 10:**
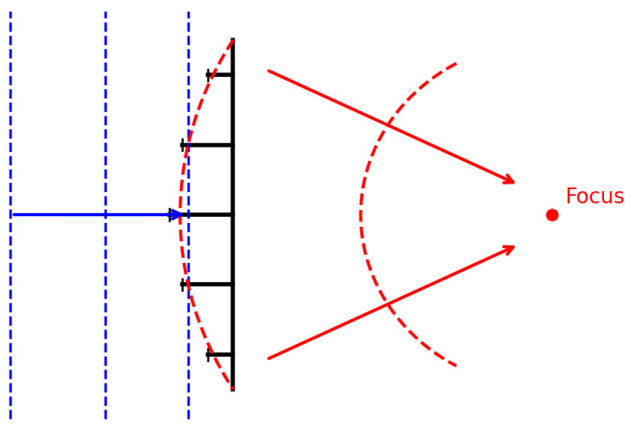
Acoustic metalenses convert plane waves into converging curved waves through phase delay design, concentrating energy at the focal point.

Acoustic metalenses are ultra-thin planar arrays of subwavelength units that focus sound by imposing a designed 0–2π phase delay across the aperture. By tailoring each unit’s geometry/material, a prescribed exit-surface phase profile is created, converting an incident plane wave into a converging curved wavefront and concentrating energy at a focal point in accordance with the generalized Snell’s law. Phase delays are commonly produced by resonant cavities (e.g., Helmholtz resonators) or by labyrinth/spiral channels that extend the propagation path. With an appropriate phase gradient, such planar single-layer lenses can achieve deep subwavelength focusing beyond the Rayleigh limit (e.g., ~λ/50 in near-field imaging), while non-resonant anisotropic designs can broaden bandwidth and reduce aberration; the subwavelength planar architecture also provides relative tolerance to incidence angle over a certain range. As shown in Figure 10, the lens structure creates a phase delay that transforms the incident plane wave into a converging wavefront, focusing the energy at the focal point [83,99,100].

In recent years, acoustic metalenses have garnered significant attention as planar focusing devices based on metamaterials, owing to their compact structure and design flexibility. By precisely manipulating sound waves through artificially engineered subwavelength units, they achieve high control over wavefront shape and propagation paths, offering novel approaches to overcoming the limitations of traditional lenses in terms of resolution, thickness, and operational frequency bands.

**Figure 11:**
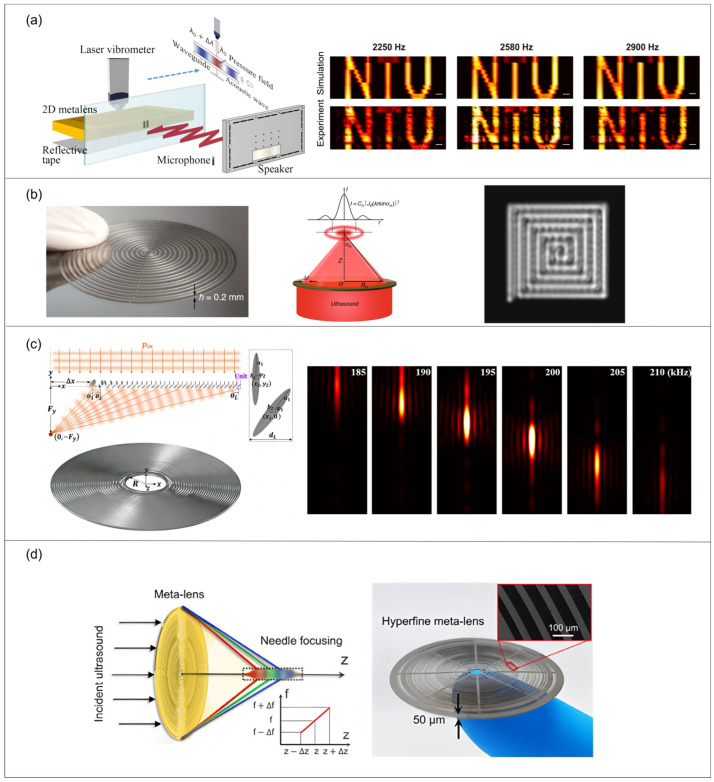
(**a**) **Left** panel shows the schematic of the experimental setup for acoustic field measurements; **right** panel shows the simulated and measured images at different frequencies: 2250 Hz, 2580 Hz, and 2900 Hz (Reprinted with permission from Ref. [102]. Copyright 2021 by the American Physical Society). (**b**) Left panel shows super-oscillation ultrasonic meta-lens; middle panel is the ultrasound image of double slits; right panel shows super-resolution imaging via a meta-lens (Reprinted with permission from Ref. [84]. Licensed under CC BY 4.0). (**c**) Schematic of the metalens, and focusing effect of the metalens at different frequencies (Reprinted with permission from Ref. [105]. Copyright 2023 American Physical Society). (**d**) The filtering effect of a metalens and dispersion for the needle-like focusing of a metalens, which can be utilized for the frequency-domain reconstruction (Reprinted with permission from Ref. [107]. Licensed under CC BY 4.0).

Within the near-field imaging paradigm, acoustic metal lenses have progressively advanced in functionality, contrast, and bandwidth. Functionally, Peng et al. [101] introduced biaxially anisotropic multilayer metal lenses, establishing two independent diffraction-free pathways to achieve image duplication, merging, and separation with sub-λ/30 resolution. Regarding performance, Chen et al. [102] enhanced the field strength within channels by employing deep subwavelength perforated metal plates, as shown in Figure 11a, achieving a lateral resolution below λ/100 while simultaneously improving contrast and expanding the bandwidth. However, this approach remains reliant on high-fabrication precision and loss control.

To overcome limitations in near-field working distances, research shifted towards far-field super-resolution strategies. In 2019, Shen et al. [84] proposed superoscillatory acoustic metasurfaces, forming a focal spot of approximately 0.3λ at a propagation distance of about 5.2λ, as shown in Figure 11b, demonstrating the feasibility of deep subwavelength focusing in the far field. However, this approach exhibited low main-beam energy and pronounced sidelobes. Subsequently, in 2022, Zeng et al. [103] compressed the far-field main lobe to approximately 0.93λ through aperture-shape engineering, successfully applying it to subwavelength crack detection. To balance resolution and efficiency, Zheng et al. [77] proposed phase-encoded acoustic metal lenses in 2022, achieving a 0.326λ focal spot and over 90% transmission in water. However, structural fabrication and multi-frequency control remain challenging. Furthermore, in 2023, as shown in Figure 11c, Fan et al. [104] proposed a ring-shaped superlattice structure, enabling axial tunable focusing while maintaining lateral resolution (FWHM ≈ 0.409λ). In the latest research, Liu et al. [105] achieved a superior balance between resolution, sidelobe suppression, and energy efficiency through dual-valued phase encoding and weighted optimization, realizing a focusing performance of approximately 0.42λ/NA with adjustable focal length.

Furthermore, Guild et al. [106] demonstrated that multiplexed acoustic vortex sources theoretically possess exceptional resolution potential, though they remain constrained by bandwidth and mode purity. In 2025, as shown in Figure 11d, Li et al. [107] combined metal lenses with nonlinear harmonic imaging and frequency-domain reconstruction to achieve volumetric super-resolution imaging, attaining resolutions of approximately 0.37$\lambda_{0}$ and 0.2$\lambda_{0}$ at fundamental and harmonic frequencies, respectively, providing a hybrid imaging approach for acoustic super-resolution.

Overall, acoustic metal lenses have evolved from early narrowband near-field resonant structures into super-resolution imaging devices, featuring far-field capability, high efficiency, and tunable characteristics. Future research must achieve further breakthroughs in reducing structural complexity, broadening operational bandwidth, and suppressing sidelobes to support practical imaging applications.

Although metamaterial lenses can overcome diffraction limits through evanescent wave enhancement/conversion, their scalability for large-area imaging remains constrained by multiple factors in manufacturing and system implementation. First, achieving deep subwavelength resolution often requires high-density unit arrays (e.g., apertures, resonators, labyrinth structures), where an increasing aperture size leads to exponential growth in unit count across the area, significantly complicating processing cycle times and consistency control. Simultaneously, the spatial accumulation of geometric tolerances induces phase or resonance drift, degrading imaging contrast and compressing the effective bandwidth. Second, further reducing channel spacing to enhance resolution accentuates thermal viscous losses and surface roughness effects, with the actual resolution additionally being constrained by the combined limitations of loss and sensor sensitivity. Third, many experimental systems still rely on point-by-point scanning readouts (each waveguide/channel corresponds to one sampling pixel), which becomes a bottleneck for real-time imaging under large-aperture conditions. Future developments should focus on sensor arrays and structural integration, or combining architectures with computational imaging strategies. Regarding fabrication, structures in the acoustic kHz band typically operate at millimeter scales and can be produced via machining, wire cutting, or additive manufacturing. However, in aquatic/ultrasonic bands, material impedance contrast and process resolution significantly impact performance. Overall, engineering metamaterial super-resolution imaging requires synergistic optimization across multiple aspects, such as structural design, loss, bandwidth, scalable manufacturing, and readout architecture.

## 5. Mechanisms and Advantages/Disadvantages of Different Methods, and Real-Time Performance Comparison

The preceding sections have discussed the contributions of various methods to enhancing the lateral resolution of acoustic images. This chapter summarizes these approaches in Table 3, comparing and summarizing their mechanisms, advantages, and limitations, and their suitability for real-time imaging.

A comparison of Table 3 reveals that physical super-resolution and algorithmic super-resolution operate on fundamentally different principles. Physical methods leverage material structures to recover evanescent waves, thereby achieving resolutions far exceeding the diffraction limit. At the algorithmic level, resolution enhancement is achieved by optimizing signal acquisition and processing strategies, with key approaches relying on sparsity assumptions, prior models, or data-driven mappings. For instance, deep neural networks accelerate ultrasound image processing to millisecond-level speeds, achieving tens of times faster processing. In contrast, ULM methods relying on microbubble accumulation typically require imaging times measured in seconds due to massive data volumes. Table 3 clearly delineates the performance characteristics of each method, aiding readers in understanding the strengths, weaknesses, and applicable scenarios of different technical approaches.

## 6. Conclusions

This review comprehensively summarizes methods for enhancing the lateral resolution of acoustic imaging, encompassing traditional focusing techniques, transducer optimization, physically structured metamaterial lenses, and approaches based on algorithmic optimization and deep learning technologies. This paper analyzes the implementation mechanisms, advantages, and disadvantages, and real-time performance of each method, highlighting that the integrated use of physical and computational approaches represents a key direction for advancing next-generation ultra-high-resolution acoustic imaging. Overall, significant progress has been made in enhancing acoustic lateral resolution through traditional focusing techniques, transducer design optimization, algorithmic refinements, and advancements in deep learning and physical innovation. Nevertheless, multiple challenges persist. Firstly, physical limitations remain formidable: constrained by the diffraction limit of sound waves, current solutions—including those employing metamaterials—can surpass lateral resolution thresholds but often suffer from narrow bandwidth, restricted field coverage, and compromised longitudinal resolution. Secondly, the complexity of algorithms and models: methods such as compressed sensing and MUSIC require computationally intensive operations, limiting real-time applicability; deep neural networks demand substantial training data and remain susceptible to scene variations. While ultrasound-guided imaging microscopy offers exceptionally high theoretical resolution, its reliance on extremely high frame rates and microbubble density results in slow imaging speeds, requiring several minutes or longer. Future research trends will inevitably involve multidisciplinary integration and multi-technique convergence. First, for real-time super-resolution imaging, the ‘high-speed’ approach should be prioritized; leveraging GPU/CUDA parallel acceleration for beamforming and post-processing, while integrating techniques, such as network pruning and quantization to reduce inference overhead, will help to alleviate computational and frame-rate bottlenecks. Second, introducing global modeling architectures, such as Swin Transformer, could enhance detail recovery capabilities, incorporating physical prior constraints to improve reliability. Concurrently, model complexity and data requirements should be controlled through lightweight design or knowledge distillation. Additionally, self-supervised or unsupervised super-resolution learning methods should be explored. In scenarios lacking paired data, reconstruction should leverage recurrent consistency frameworks like CycleGAN, while multi-scale losses and physical constraints should be employed to improve training stability and cross-domain robustness. At the physical level, the development of broadband metamaterial focusing technologies should continue. Meanwhile, the use of multi-resonant or superlattice structures will expand the operational bandwidth while suppressing mutual coupling and reducing manufacturing sensitivity through topological optimization and multiphysics simulations. The integration machine-learning-assisted inverse design for metamaterials will enable the use of generative or surrogate models to accelerate the “simulation-optimization” closed loop and minimize manual intervention. Finally, the development of programmable active acoustic metamaterials will enable dynamically tunable focusing, providing a reconfigurable hardware foundation for synergistic metamaterial computational imaging. Collectively, these pathways refine challenges and propose actionable solutions across three main dimensions: ‘computational power and real-time performance, models and data, and materials and system integration’, outlining clear research directions for achieving high-resolution, generalizable, and real-time next-generation acoustic imaging.

## Figures and Tables

**Table 2:** Architecture and illustrative diagram of GAN networks and their enhanced models for acoustic super-resolution imaging.

	Mishra et al. [64]	Nair et al. [65]	Wang et al. [66]	Liu et al. [69]	Ding et al. [70]	Si et al. [71]
**Model Architecture**	GAN-based Despeckling Residual Neural Network (DRNN)	GAN	Conditional GAN (cGAN)	CycleGAN (self-supervised)	Two-Stage CycleGAN	CycleGAN (RADD modification)
**Image Type/Resolution**	Liver ultrasound images	Simulated ultrasound images (Field-II)	Ultrasound RF channel (PICMUS)	CCA-US, CCA-US datasets	Scanning acoustic microscopy (SAM)	CPWC ultrasound data
**Performance Metrics**	PSNR, SSIM, edge preservation	PSNR, DSC scores	SNR improvement, Cross-correlation	PSNR, SSIM, inference efficiency	NRMSE, PSNR	SNR, SSIM
**Image Quality Improvement**	Pre: PSNR = 27.5 dB, SSIM = 0.85, Post: PSNR = 30.2 dB, SSIM = 0.92	Pre: PSNR = 29.38 dB, DSC = 0.908, Post: PSNR = 14.86 dB, DSC = 0.79	Pre: SNR = 1.112, Post: SNR = 1.540, Cross-correlation (Pre: 0.641, Post: 0.976)	Pre: PSNR = 30.2 dB, SSIM = 0.92, Post: No paired data for training	Enhanced 180 MHz SAM image resolution	Pre: SNR = 7.8% increase, SSIM = 22.2% increase, Post: Enhanced resolution
**Loss Function**	Adversarial loss, structural loss	Adversarial loss, DNN segmentation	cGAN loss, L1 loss	Cycle-consistency loss, adversarial loss	Biological tissue imaging	CycleGAN loss, residual attention

**Table 3:** Comparison of representative methods for improving lateral resolution in acoustic imaging in terms of mechanisms, advantages, limitations, and real-time performance.

**Method**	Traditional ultrasound optimization (such as transducer design, focused probe design, and array design)	Compressed sensing	MUSIC	Deep learning	Metamaterial lens	ULM
**Mechanism**	Hardware and system-level optimization (physical focusing, broadband signals, etc.)	Signal Reconstruction Algorithm based on Sparse Priors	Multi-signal subspace-based (algorithm resolution enhancement)	Enhanced computational resolution for neural network models	Physical focusing based on metamaterial negative refraction or waveguide structures	Microbubble tracking, computational method for super-resolution imaging
**Advantages**	Maintain the same real-time capability as conventional imaging; easy to implement	Reduce the sampling volume and transmission frequency, accelerating the acquisition speed.	Ultra-high resolution (capable of locating subwavelength targets)	Restores high-frequency details, significantly enhances resolution, and effectively suppresses noise.	Physically overcomes the diffraction limit, enabling amplification and transmission of evanescent waves without post-processing.	Significant resolution enhancement (up to approximately 10 times the standard resolution)
**Limitations**	Resolution improvement is limited due to diffraction-limited constraints; increasing pulse count or reducing frame rate is required	Sensitive to noise; reduced signal-to-noise ratio; computationally intensive iterative solution process	Requires prior estimation of scatterer counts; sensitive to model errors and noise; computational complexity	Requires large amounts of labeled training data; prone to generating artifacts; limited model transferability and interpretability; time-consuming model training	Narrow operating bandwidth (effective only at specific frequencies); complex manufacturing; inherent losses; stringent application conditions	Contrast agent injection is required; acquisition time is extremely long
**Real-time performance**	Real-time imaging capability (frame rate depends on the composite angle)	Processing is slower and typically cannot provide real-time imaging	Typically requires a long time to compute, resulting in poor real-time performance	Improved resolution and textural quality	Currently, most applications originate from experimental settings; prospects for expanding into industrial and clinical real-time imaging remain limited	Current processing is primarily offline, far below real-time standards

## Data Availability

No new data were created or analyzed in this study.
